# Emotion Recognition Using Temporal Facial Skin Temperature and Eye-Opening Degree During Digital Content Viewing for Japanese Older Adults

**DOI:** 10.3390/s25216545

**Published:** 2025-10-24

**Authors:** Rio Tanabe, Ryota Kikuchi, Min Zou, Kenji Suehiro, Nobuaki Takahashi, Hiroki Saito, Takuya Kobayashi, Hisami Satake, Naoko Sato, Yoichi Kageyama

**Affiliations:** 1 Department of Informatics and Data Science, Faculty of Informatics and Data Science, Akita University (Tegata Campus), 1-1 Tegata Gakuen-machi, Akita-shi 010-8502, Akita, Japan; rrr54twork@gmail.com (R.T.); kikuchir@gipc.akita-u.ac.jp (R.K.); 2 Computer and Information Science Course, Faculty of Science and Engineering, Iwate University (Ueda Campus), 4-3-5 Ueda, Morioka-shi 020-8550, Iwate, Japan; zou@iwate-u.ac.jp; 3 Cable Networks Akita Co., Ltd., 1-3 Yabase Minami-1tyoume, Akita-shi 010-0976, Akita, Japan; suehiro@cna-catv.co.jp (K.S.); nobuaki-t@cna-catv.co.jp (N.T.); h-saito@cna-catv.co.jp (H.S.); tak-koba@cna-catv.co.jp (T.K.); 4 ALL-A Co., Ltd., 1-3 Yabase Minami-1tyoume, Akita-shi 010-0976, Akita, Japan; hisami-s@all-a.jp (H.S.); naoko.sato@all-a.jp (N.S.)

**Keywords:** emotion recognition, skin temperature, eye opening, aging population, digital content, healthy life expectancy, Japanese older adults

## Abstract

Electroencephalography is a widely used method for emotion recognition. However, it requires specialized equipment, leading to high costs. Additionally, attaching devices to the body during such procedures may cause physical and psychological stress to participants. These issues are addressed in this study by focusing on physiological signals that are noninvasive and contact-free, and a generalized method for estimating emotions is developed. Specifically, the facial skin temperature and eye-opening degree of participants captured via infrared thermography and visible cameras are utilized, and emotional states are estimated while Japanese older adults view digital content. Emotional responses while viewing digital content are often subtle and dynamic. Additionally, various emotions occur during such situations, both positive and negative. Fluctuations in facial skin temperature and eye-opening degree reflect activities in the autonomic nervous system. In particular, expressing emotions through facial expressions is difficult for older adults; as such, emotional estimation using such ecological information is required. Our study results demonstrated that focusing on skin temperature changes and eye movements during emotional arousal and non-arousal using bidirectional long short-term memory yields an F1 score of 92.21%. The findings of this study can enhance emotion recognition in digital content, improving user experience and the evaluation of digital content.

## 1. Introduction

Emotion plays a crucial role in human decision-making, behavior, and communication [1,2,3]. As a result, the demand for automated emotion-recognition systems has surged, making it a rapidly advancing field of research. Despite significant progress, accurately identifying human emotions and leveraging them for decision-making and action remains a challenging task for computers [4,5]. Older adults often display emotions differently from younger individuals because of factors such as age-related changes in facial muscles and cognitive impairments associated with dementia, making emotion recognition more challenging. In recent years, the proportion of older adults in the global population has been steadily increasing, making aging a global trend [6]. Consequently, there is an urgent need to develop effective emotion-recognition systems for older adults. As an example, an empirical study has been conducted to examine the potential enhancement of cognitive functions through emotion recognition during gameplay targeting Japanese older adults [7].

If successful, emotion recognition can be applied across various domains, including healthcare [8,9,10,11], education [12], and marketing [13], in addition to more specialized areas, such as public transportation congestion management [14] and driver assistance systems [15]. Moreover, emotion recognition can serve as a key indicator for assessing user engagement with digital content and overall viewing quality.

Ekman et al. [16] stated that emotions can be classified into six basic categories. Russell’s circumplex model [17] further classifies emotions along two key dimensions: arousal and valence. Valence reflects the intensity of the positive or negative feeling associated with an emotion, whereas arousal represents the degree of physical and cognitive activation, ranging from calm to excited states, independent of emotional valence. However, one limitation of this approach is its inability to effectively differentiate between emotional arousal intervals—periods where emotions are heightened—and emotional non-arousal intervals—periods where emotions are subdued. Moreover, research addressing the discrimination between these two intervals remains insufficient. Therefore, further exploration is needed to develop methods that can more accurately distinguish between emotional arousal intervals and non-arousal intervals. To address this gap, in this study, we focus on distinguishing between emotional arousal and non-arousal intervals. The rationale for this approach lies in the fact that the authors of prior studies have not adequately explored the distinction between these two conditions. In particular, although numerous studies have examined diverse emotional categories and valence dimensions, investigations into non-arousal intervals remain relatively scarce.

Human communication is mediated by various types of information, including speech, facial expressions, and gestures. Facial expressions, in particular, are essential for conveying emotional content, as they transmit a substantial amount of information through movements of different facial features, such as the eyes, mouth, and cheeks. Birdwhistell et al. [18] reported that verbal communication accounts for only 35% of all communication; in comparison, nonverbal cues—such as facial expressions and gestures—constitute the remaining 65%.

In the context of emotion recognition from facial expressions, Almeida et al. [19] proposed an emotion-recognition method based on a convolutional neural network (CNN) to investigate the applicability of current automatic emotion-recognition systems in the film industry. The method includes the use of the FER2013 [20] and SFEW [21] datasets, which contain facial expressions triggered by videos. Manalu et al. [22] introduced a custom CNN–recurrent neural network (RNN) model for recognizing facial expressions in videos via the Emognition Wearable Dataset 2020 [23], which includes emotions such as awe, amusement, liking, anger, enthusiasm, disgust, neutral, sadness, surprise, and fear. However, natural facial expressions associated with emotions can vary significantly across individuals. Furthermore, humans possess the ability to consciously control their facial expressions and vocal tones [24]. Therefore, investigating physiological indicators, such as changes in facial skin temperature, which are independent of voluntary facial expressions, and integrating these measures with facial expression analysis are crucial for a more comprehensive understanding of emotional responses.

Physiological signals commonly employed for emotion recognition include electrocardiogram (ECG) [25], electromyogram (EMG) [26], skin temperature [27], electroencephalography (EEG) [28], photoplethysmography [29], heart rate (HR) [30], and galvanic skin response (GSR) [31]. Among these methods, contact information such as EEG is particularly prominent as a widely used method for emotion recognition [32]. As recent examples of EEG-based studies, Hu et al. [33] proposed STRFLNet, a deep learning model that integrates graph-based structures and transformer modules to fuse spatio-temporal features from contact-based EEG signals, aiming to enhance the accuracy of emotion recognition. The model demonstrated superior performance compared to existing methods across multiple public EEG datasets under both subject-dependent and subject-independent settings. Kachare et al. [34] proposed STEADYNet, a CNN-based architecture that processes multichannel temporal EEG signals to extract spatiotemporal features for automatic dementia classification. The model achieved high accuracy across multiple dementia types. Liu et al. [35] conducted a comprehensive review of EEG-based multimodal emotion recognition (EMER), focusing on machine learning approaches for feature representation, signal fusion, and incomplete modality handling. Their work addresses methodological gaps in prior reviews by emphasizing EEG as the central modality.

For emotion recognition via contact information, Pradhan et al. [36] proposed the HEPLM approach for the WESAD [37] dataset, which includes ECG, electrodermal activity (EDA), EMG, respiration (RSP), and skin temperature data collected from 17 participants. Jaswal et al. [38] proposed a CNN model to classify three emotions (happiness, sadness, and neutral) by selecting features from EEG and mel frequency cepstral coefficient (MFCC) signals via the gray wolf optimization (GWO) algorithm [39]. However, signals such as ECG, EEG, and GSR require specialized equipment, which often incurs high costs and may introduce physical and psychological stress for participants owing to the necessity of contact-based measurements. Therefore, noncontact information, which can be easily obtained without placing additional stress on the participants, is crucial. In response to these limitations, in our study, we employ non-contact physiological signals that minimize physical and psychological stress, particularly for Japanese older adults, and enhance feasibility in real-world applications.

With respect to emotion recognition via noncontact information, Zhang et al. [40] proposed both unimodal and multimodal models using a Mask region-based CNN (R-CNN) model that incorporates HR data (noncontact), eye movements, and facial expressions. Chatterjee et al. [41] introduced a method that employs the MobileNet model for training and the Grunwald–Letnikov Moth Flame Optimization (GL-MFO) algorithm for feature optimization, targeting the Thermal Face Database [42], which includes facial thermal images with deliberate expressions and emotions from participants not wearing glasses. Among these methods, the study authors focus on facial skin temperature data obtained from infrared thermography (IRT) and facial expressions captured through a visible camera.

The authors of previous studies on facial skin temperature have focused on observing changes in the temperature of the nose and cheek areas during emotional arousal, particularly in response to joy. A facial detection method that combines both thermal and visible images has been proposed to investigate the correlation between joy and specific regions of interest (ROIs) [43]. Additionally, emotion-recognition studies involving the use of CNNs with both thermal and visible facial images have demonstrated superior performance when thermal images are used over visible images [41,44,45]. In one study [41], an accuracy of 97.47% was achieved across seven emotion categories: anger, disgust, fear, happiness, sadness, surprise, and neutral (emotional non-arousal interval). Furthermore, changes in eye state have been identified as key indicators for estimating emotion and pain [46], and their integration has been shown to significantly improve the accuracy of emotion classification [40,47]. Moreover, the authors of a previous study [31] demonstrated that multimodal models, combining multiple methods and features, are more effective for emotion recognition than unimodal models that rely on a single feature. Despite these advancements, the combination of thermal and visible images, particularly those that integrate changes in skin temperature with eye state variations, remains underexplored.

The authors of previous studies have focused on analyzing changes in the skin temperature of the nose, as well as the right and left cheek regions, alongside the relative positional information of facial features during emotional arousal, particularly in response to joy. Additionally, methods for estimating emotional arousal intervals have been investigated [48]. Significant changes in skin temperature and facial movements have been observed during joyful arousal intervals. Furthermore, the feasibility of estimating emotional arousal intervals via machine learning techniques has been demonstrated. However, our previous model was limited to joy and lacked generalizability across other emotions. Therefore, in this study, skin temperature variations (in the nose, right cheek, and left cheek areas) and the relative positions of facial features (eyes and mouth) were analyzed across multiple emotions. Significant differences were verified between the emotional arousal and non-arousal conditions. Moreover, methods for estimating these emotions have been further examined [49,50]. The results reveal notable differences in skin temperature changes and eye-opening/closing patterns. Furthermore, emotional arousal and non-arousal intervals can be estimated via machine learning, which achieves accuracies exceeding 80% when these features are combined. However, accuracy was reduced for some participants, with results falling to approximately 60%.

The issues associated with emotion recognition in the previously discussed studies are summarized as follows:

1. In many studies, emotions are represented via arousal and valence dimensions, making it difficult to evaluate whether emotional arousal and emotional non-arousal intervals can be accurately distinguished. Additionally, various methods have been developed to classify multiple emotions and neutral (defined as emotional non-arousal in this study). For accurate emotion estimation, it is crucial to determine whether an emotion is present or absent. However, these studies do not focus on distinguishing between emotional arousal and emotional non-arousal intervals.
2. In recent years, the proportion of older adults in the global population has been steadily increasing, making aging a global trend. However, the datasets used in existing emotion-recognition research have primarily been limited to younger individuals, and datasets specifically targeting older adults have not been adequately explored.
3. Research findings have demonstrated the significance of skin temperature variations and eye information in emotion recognition. Furthermore, multimodal approaches that utilize multiple methods and features outperform unimodal approaches that rely on a single feature. However, the combination of thermal and visible images, particularly those that integrate changes in skin temperature with eye state variations, remains underexplored.

The aim of this study is to develop a generalized methodology for estimating emotional arousal intervals, focusing on time-series data of skin temperature changes in the nose and cheek regions and the distance between the upper and lower eyelids (eye-opening degree (EOD)) during emotional arousal while participants view digital content. A method for distinguishing between emotional arousal and non-arousal intervals is proposed, and the threshold for distinguishing these intervals is investigated. Additionally, as emotional arousal intervals are continuous, a correction method based on the recognition results from preceding and succeeding frames is explored. Comparisons are made with previous studies to evaluate the robustness of the proposed method. For the purpose of comparison, the distinction between emotional arousal and non-arousal intervals must be evaluated on the basis of the confusion matrix presented in the literature by grouping positive and negative emotions, or multiple emotions, as emotional arousal intervals. Therefore, the related works for comparison should include studies that classify multiple emotions and those that distinguish between positive emotions, negative emotions, and emotional non-arousal intervals. Related literature that meets these criteria, together with their characteristics, is presented in Table 1. A related work [41] that focuses on participants without glasses and employs deliberately induced facial expressions and emotions is excluded from the comparison. Thus, the related works for comparison include references [19,22,36,38,40].

The primary contributions of this study are as follows.

1. A novel emotion-recognition method that utilizes skin temperature and EOD to distinguish between emotional arousal and non-arousal states is proposed.
2. The effectiveness of combining thermal and visible images, particularly those focusing on skin temperature changes and EOD, is demonstrated.
3. The effectiveness of noncontact features for emotion recognition is assessed.
4. The proposed method is compared with existing emotion-recognition methods that are based on noncontact features.
5. The effectiveness of a method that automatically sets thresholds for classifying emotional arousal and non-arousal based on individual participants and corrects results by preceding and subsequent estimates in improving emotion-recognition accuracy is examined.

The remainder of the paper is organized as follows: In Section 2, we describe the data-acquisition environment and procedures, in Section 3, we present the proposed emotion-recognition methodology, in Section 4, we present a discussion on the results of the proposed methodology, and in Section 5, we conclude the paper and outline future directions.

## 2. Data Acquisition

In a related study [19], the authors reported on an emotion-recognition method using digital content presented to participants that facilitates the capture of naturally occurring facial expressions and emotions. Building on this approach, the following digital content was presented to the participants in this study: a documentary capturing behind-the-scenes footage of the Omagari Fireworks Festival in Akita, which was canceled because of the COVID-19 pandemic, with a runtime of 27 min and 43 s. The existing datasets used for emotion recognition are primarily limited to younger participants, with insufficient consideration given to datasets centered on older adults. To fill this gap, we focus on Japanese older adults as our target population in the present study.

To acquire data for this study, facial thermal images (640 × 480 pixels, 30 fps) and visible images (1920 × 1080 pixels, 60 fps) were captured from nine participants (A–I: two males and seven females, aged 60–80, Asian) while viewing the digital content. The thermal images were captured using IRT devices (R500EX-S, R550S, Nippon Avionics Co., Ltd., Yokohama, Japan) [51,52], and the visible images were captured using a 4K video camera (HC-VX2M, Panasonic Co., Ltd., Tokyo, Japan) [53]. During the experiment, participants were asked to rate the types, intervals, and intensities of emotions they experienced while viewing the digital content. Intensity was rated on three levels: strong, medium, and weak.

**Figure 1:**
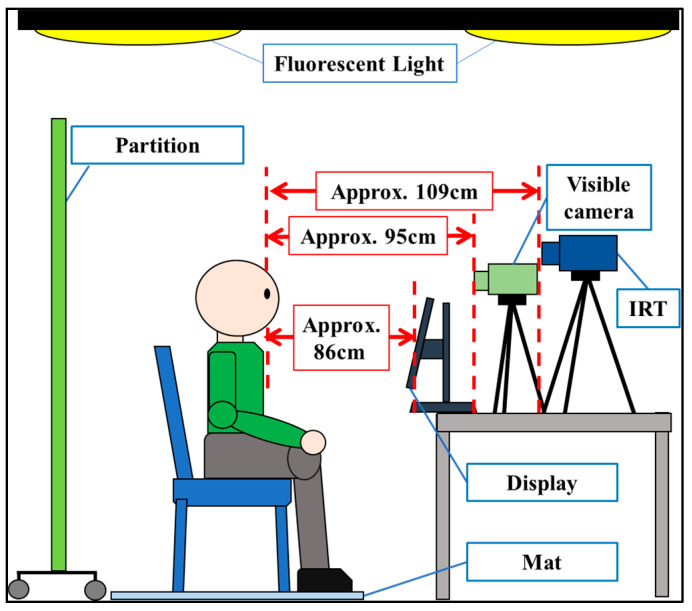
Data-acquisition environment.

The data-acquisition environment is depicted in Figure 1. A resting period of approximately five minutes was provided before data acquisition to minimize the influence of tension and other external factors. The participants were not given a predefined list of emotions; rather, they were encouraged to freely express the emotions they experienced. In this study, “strong” emotions were classified as emotional arousal intervals, whereas intervals that were not rated were classified as emotional non-arousal intervals. Table 2 presents the types of emotions observed in this research. The acquisition conditions for the thermal and visible facial videos during data collection are detailed below.

- Room temperature: 21.4–26.4 °C
- Humidity: 48.2–69.5%
- Illumination (above the participant): 703–889 lx
- Illumination (front of the participant): 255–361 lx

The data used in this study were acquired in accordance with ethical regulations for human research at Akita University, Japan, with approval granted on 12 March 2021. Informed consent was obtained from all participants included in the study. The data were collected over two days, on 6 November and 7 November 2023. Additionally, appropriate countermeasures were implemented to ensure that adequate precautions were taken against COVID-19 infection during data acquisition.

## 3. Proposed Methodology

### 3.1. Face Detection Method

**Figure 2:**
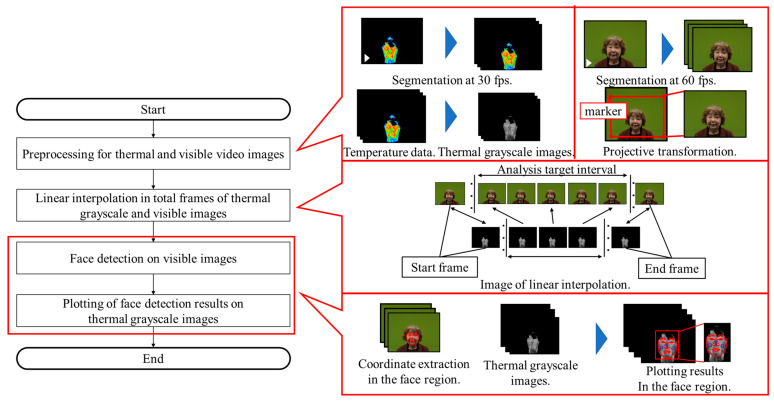
Flow of the face detection method.

To examine in detail the relationship between emotional arousal and temperature changes in the ROIs, it is essential to analyze the temperature variations over time within these regions. The facial detection method [49,50], which integrates thermal and visual video images, is illustrated in Figure 2. This method is described in the following Section 3.1.1, Section 3.1.2, Section 3.1.3, Section 3.1.4.

#### 3.1.1. Preprocessing of Thermal and Visible Video Images

First, the thermal video image was segmented at 30 fps. Next, a grayscale image (thermal grayscale image) was generated from the temperature data from the thermal video image. The temperature range of 29.0–37.0 °C was normalized to correspond to luminance values ranging from 0 to 255. The visible video image was subsequently segmented at 60 fps. Lastly, a projective transformation was applied to the area defined by the four markers, transforming it to a resolution of 640 × 480 pixels and matching the thermal grayscale image.

#### 3.1.2. Linear Interpolation in Total Frames of Thermal Grayscale and Visible Images

The total number of frames in the thermal grayscale image and the visible image differ owing to the use of two distinct cameras for image capture. As a result, the number of frames was linearly interpolated to determine the thermal grayscale image frame that corresponds temporally to an arbitrary frame in the visible image.

#### 3.1.3. Face Detection on Visible Images

The face detection function from the open-source library insightface [54] was employed to obtain the facial area coordinates for frames within the interval to be analyzed in the visible video image. A total of 106 facial area coordinates were obtained through face detection.

#### 3.1.4. Plotting of Face Detection Results on Thermal Grayscale Images

The facial area coordinates from the visible video image, acquired using insightface, were overlaid onto the corresponding thermal grayscale image, ensuring temporal alignment between the two images.

### 3.2. Setting ROIs

**Figure 3:**
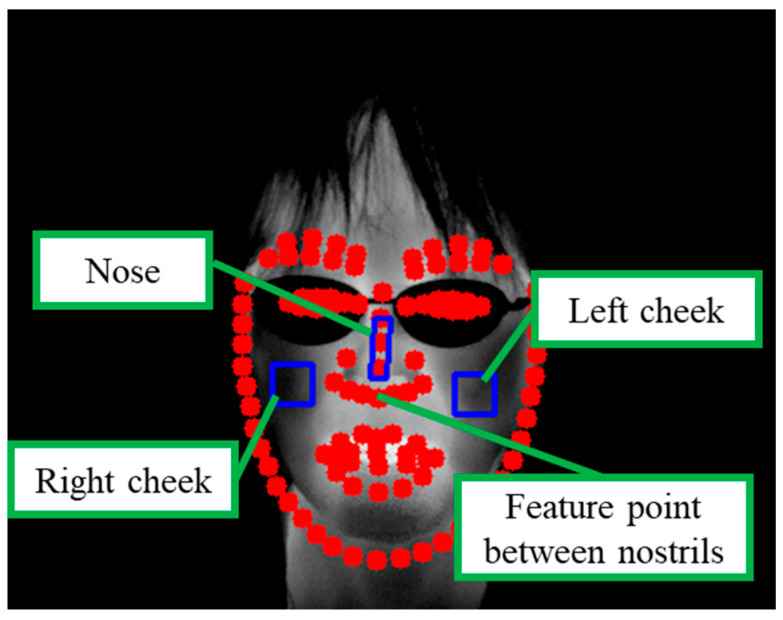
Example of setting ROIs (The red dot shows the facial area coordinate).

The ROIs are defined on the thermal grayscale image after face detection. The conditions for setting the ROIs are described below. Figure 3 provides an example of the ROI setup. The tilt of a participant’s face may change during the viewing of digital content. As illustrated in Figure 3, the reference feature point between the nostrils was used to account for facial tilt, and the ROIs were rotated accordingly to align with the facial orientation.

- Nose: Area between the apex and root of the nose, excluding the nostrils (10 × 30 pixels);
- Cheeks: Area below the eyes, excluding the eyes, nose, and mouth (20 × 20 pixels).

### 3.3. Feature Extraction

For practical applications, detecting and estimating arousal intervals via machine learning requires fast processing. Directly using luminance values from thermal images as training data can potentially accelerate processing compared with measuring skin temperatures with IRT. In this section, we outline a method for extracting luminance values from the ROIs in thermal grayscale images. The method calculates the luminance temperature (LT) on the basis of the average luminance value and the LT difference between ROIs and applies a smoothing process to the LT to reduce noise. Furthermore, the temperature change (amount of temperature change (ATC)) is calculated by obtaining the difference from the previous second in the time-series data. Furthermore, the method for calculating the EOD is described.

#### 3.3.1. LT

First, luminance values were obtained from the ROIs in the thermal grayscale images. The means of these values were then computed. Thereafter, the LT was calculated using Equation (1). LT enables temperature changes to be detected over time. Here, *T*_*color*_ represents the LT, *G*_*avg*_ denotes the average luminance values, and *T*_*max*_ and *T*_*min*_ represent the highest and lowest temperatures within the normalization temperature range, respectively.

(1) $$\;T_{color\;}=G_{avg}/255\left(T_{max}-T_{min}\right)+T_{min}$$

#### 3.3.2. LT Difference

The difference in LT between the ROIs was calculated. The ROIs for which the LT differences are computed are as follows. The LT difference allows for the detection of relationships between these ROIs.

- Nose and right cheek;
- Nose and left cheek;
- Right and left cheeks.

#### 3.3.3. ATC

First, a smoothing process was applied to the LT to reduce noise. Specifically, a moving-average filter was applied to the target frame, in addition to one frame before and one frame after the target frame. The difference between the temperature in the frame of interest and the frame 30 frames prior (equivalent to one second) was subsequently calculated, resulting in the ATC for the smoothed time-series data. The ATC enables the detection of short-term temperature changes.

#### 3.3.4. EOD

**Figure 4:**
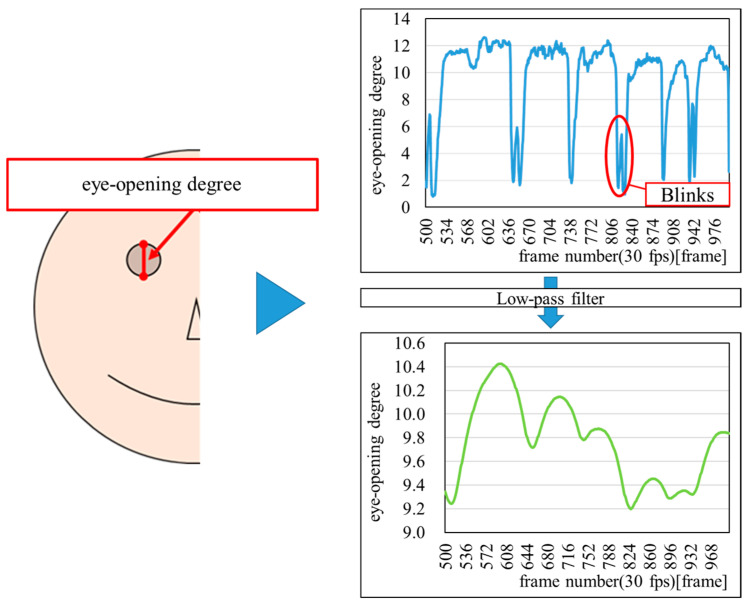
Example of the result of calculating the eye-opening degree (EOD).

First, the EOD was calculated as the distance between the upper and lower eyelids, based on face area coordinates obtained using insightface. Next, a low-pass filter was applied to pass low-frequency components while attenuating high-frequency components, effectively excluding blink-related noise. Figure 4 shows an example of the result of calculating the EOD.

### 3.4. Emotion Recognition

**Figure 5:**
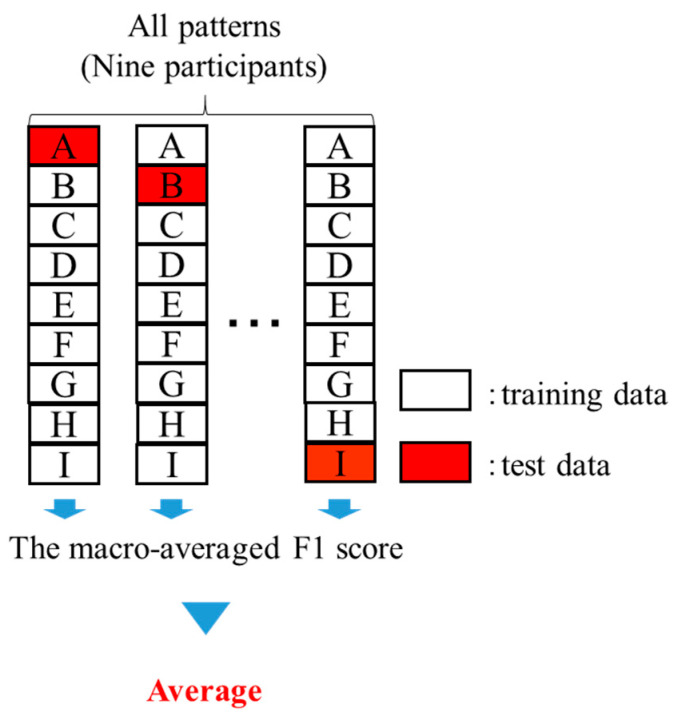
Flow of cross-validation.

Classification was performed by assigning a label of zero to the emotional arousal intervals and one to the emotional non-arousal intervals. First, the features calculated in Section 3.3 were input into a bidirectional long short-term memory (BLSTM) model [55], consisting of three layers—an input layer, an intermediate layer, and an output layer—to process the time-series data. Next, cross-validation was performed using data from all nine participants. In each iteration, one participant was withheld from the training set and used as the test data. The macro-average F1 score was then calculated for each model, and the success rate of estimating emotional arousal and non-arousal intervals was evaluated by averaging the macro-average F1 scores across all iterations. Figure 5 illustrates the flow of the cross-validation process. The optimal hyperparameters were determined through preliminary experiments, and these hyperparameters are listed in Table 3.

#### 3.4.1. BLSTM

**Figure 6:**
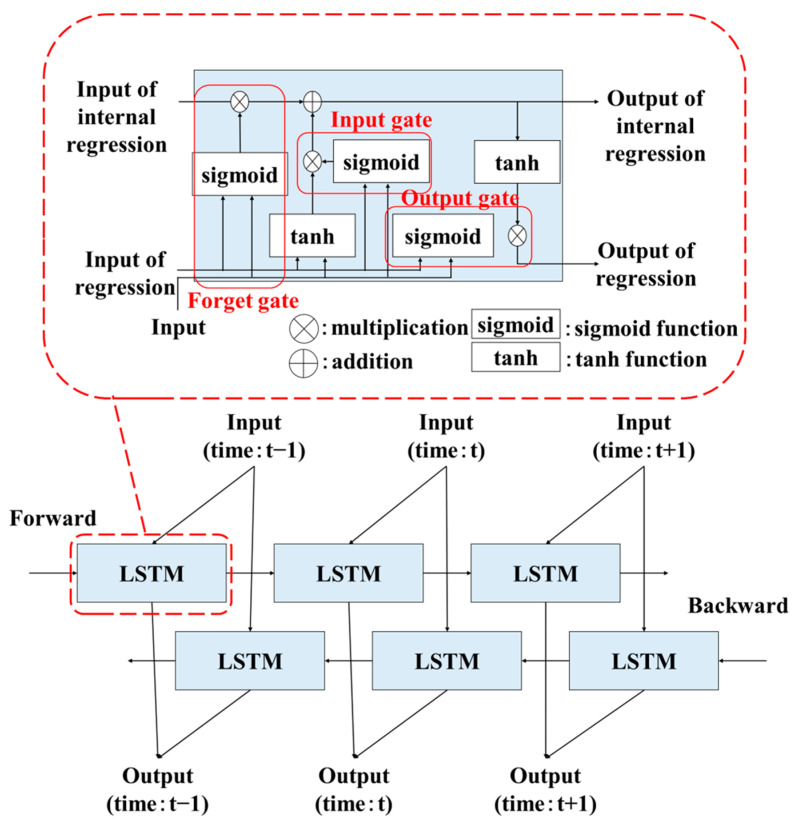
Flow of processing time-series data using BLSTM and its internal structure.

The BLSTM model is specifically designed for processing time-series data by simultaneously processing inputs in both forward (past) and backward (future) directions. In addition to regression, it includes an internal regression that is completed within a single cell. Additionally, it comprises an input gate, a forget gate, and an output gate. These three gates are used to control the information in the internal regression. Figure 6 illustrates the flow of processing time-series data using BLSTM and its internal structure.

#### 3.4.2. Classification Threshold

**Figure 7:**
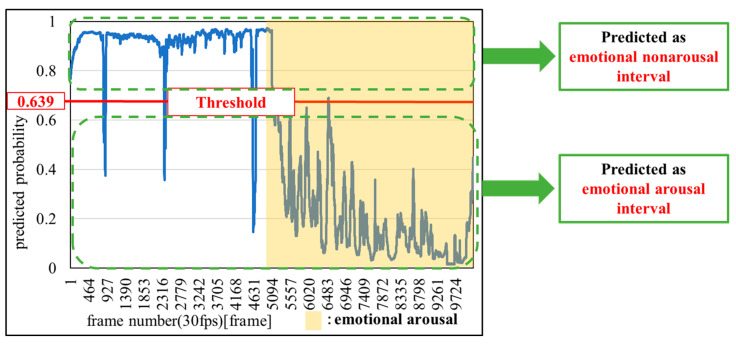
Example of setting a classification threshold.

Figure 7 shows an example of setting a classification threshold. In this study, the median value of the blue waveform in Figure 7, which represents the predicted probability from the machine learning model, was used as the threshold for classifying the intervals. If the predicted probability was closer to one than the threshold, the interval was classified as an emotional non-arousal interval; conversely, if the value was closer to zero, the interval was classified as an emotional arousal interval.

### 3.5. Performance Metrics

In this study, we evaluate the emotion-recognition method using the macro-average F1 score, which is the average of the F1 scores [57] for the emotional arousal interval and the emotional non-arousal interval. First, the F1 scores for the emotional arousal interval (class: zero) and the emotional non-arousal interval (class: one) were calculated using Equation (2). The macro-average F1 score, which is the mean of the F1 scores, was subsequently computed. The macro-average F1 score ranges from 0.0 to 1.0. The closer the value is to 1.0, the higher the success rate in estimating the emotional arousal interval and the emotional non-arousal interval.

(2) $$F1\;score=\frac{TP}{TP+\frac{1}{2}\left(FP+FN\right)}\times 100$$

where *TP*, *FP*, and *FN* represent true positives, false positives, and false negatives, respectively.

### 3.6. Emotion Recognition

**Figure 8:**
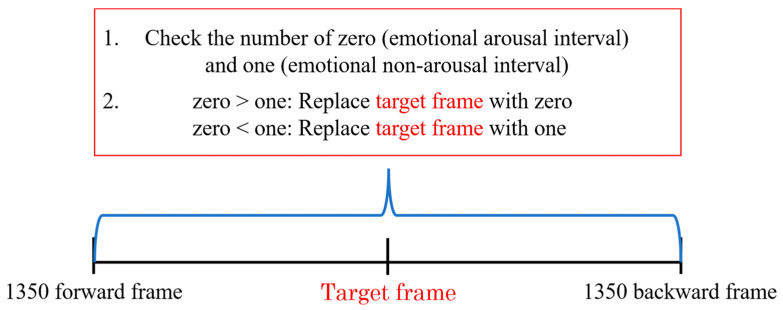
Illustration of the correction method.

To reduce misclassification, a correction method was applied based on the estimation results from both forward and backward frames, with a focus on the fact that the emotional arousal interval is a sequence of frames. Specifically, several forward and backward frames surrounding the target frame were examined, and correction was performed using the most frequent labels. Figure 8 illustrates the correction method.

Dr. Jill Bolte Taylor, a neuroscientist at Harvard University, proposed the 90 s rule, asserting that the emergence of emotions and accompanying physiological changes follow a specific pattern [58]. Based on this rule, emotional responses (including physiological changes) last for 90 s. Thereafter, any lingering emotional response is merely the result of the individual choosing to remain in the emotional loop. Therefore, the correction was applied within a 90 s window (1350 forward and backward frames, totaling 2700 frames).

## 4. Analysis of Results and Discussion

Table 4 presents the macro-average F1 scores for each participant assigned to the test data. The proposed method achieved an average value of 92.21%, exceeding 80.00% for all participants. This finding demonstrates that the proposed emotion-recognition method can successfully discriminate between emotional arousal intervals and non-arousal intervals with an average accuracy of over 92.21%. Additionally, the method proved effective for estimating emotional arousal in previously unseen participants. Consequently, the findings of this research contribute to advancing emotion recognition in digital content, improving the user experience, and enhancing the evaluation of digital content.

To assess the effectiveness of the proposed method, a comparison was conducted with the accuracies of related studies [19,22,36,38,40], as discussed in Section 1. Some related studies were designed to classify multiple emotions and distinguish between positive and negative emotions, in addition to emotional non-arousal intervals. Therefore, on the basis of the provided confusion matrix, positive and negative emotions, combined with multiple emotional categories, were grouped as emotional arousal intervals. The macro-average F1 score for the emotional arousal and non-arousal intervals was calculated and compared across various models. The comparison results for the proposed method are presented in Table 5. The proposed method outperforms models that rely solely on facial expressions. Additionally, the proposed method is more accurate than the models that incorporate HR (noncontact), eye movements, and facial expressions, in addition to multimodal models that combine these features.

The accuracy of the proposed model is lower than those of the models that utilize EEG and other features obtained through contact-type devices. Despite this, contact-type devices may induce psychological and physical stress in participants during data collection. Furthermore, the features employed in this study can be acquired in a noncontact manner, which offers the distinct advantages of being suitable for online use and being easily scaled for multiple participants. Consequently, the proposed method is more advantageous in terms of its practical utility and applicability.

## 5. Conclusions

The aim of this study was to develop a generalized methodology for estimating emotional arousal intervals and distinguishing between emotional arousal and emotional non-arousal states, focusing on time-series data of skin temperature changes in the nose and cheek regions and the distance between the upper and lower eyelids (EOD), during emotional arousal while participants viewed digital content. To evaluate the robustness of the proposed method, comparisons were performed with related studies. Specifically, approaches for classifying multiple emotions or distinguishing between positive and negative emotions, as well as emotional non-arousal intervals, have been adopted in certain studies. On the basis of the provided confusion matrix, positive and negative emotions, along with multiple emotional categories, were grouped as emotional arousal intervals. The macro-average F1 score for the emotional arousal and non-arousal intervals was then calculated and compared across various models.

The conclusions of this study are summarized as follows:

1. The proposed method achieves an accuracy of 92.21% in classifying emotional arousal and non-arousal intervals.
2. The proposed method outperforms existing emotion-recognition methods that rely on noncontact information.
3. The proposed method effectively integrates thermal and visible images, providing enhanced recognition performance.
4. The proposed method highlights the significance of skin temperature variations and eye openness in emotion recognition.

A limitation of this study is the relatively small sample size. In future studies, we will focus on expanding the participant pool and exploring the method’s effectiveness across a broader age range. In addition, future studies will include participants with more diverse backgrounds—such as varying cognitive characteristics, lifestyle habits, and cultural contexts—in order to further enhance the robustness and applicability of the findings. Additionally, the proposed method has been shown to be effective in distinguishing between emotional arousal and emotional non-arousal states compared with existing methods. However, it has not been examined in detail for individual emotions, and the correlations between different emotions remain unclear. These aspects will be explored in future studies. Specifically, the granularity of emotion classification can be enhanced by adopting a two-dimensional model, such as Russell’s circumplex model, which integrates both valence (pleasantness–unpleasantness) and arousal dimensions. This approach is expected to enable more nuanced emotion recognition and improve the applicability and robustness of the proposed framework across diverse emotional contexts. Additionally, in this study, we employed the BLSTM architecture, a well-established method for handling temporal physiological data, to assess the effectiveness of the selected features. Looking ahead, in future work, we will aim to improve both performance and originality by exploring alternative modeling strategies. Potential directions include the adoption of transformer-based temporal models, graph neural networks, and multimodal contrastive learning frameworks. Moreover, the absence of standardized emotion elicitation paradigms and objective validation methods poses a significant challenge to the reliability and reproducibility of the labeling process. To address this issue, we will adopt a more robust labeling strategy that integrates multiple sources of information, including self-reports, external annotations, and physiological indicators such as GSR and heart rate.

Ultimately, the findings of this study can significantly enhance emotion recognition in digital content, leading to improved user experiences and more effective evaluations of such content. Furthermore, this approach has the potential to help caregivers better understand the condition of Japanese older adults, enabling the provision of more personalized care. For example, in healthcare and digital wellness platforms, non-contact emotion recognition could be integrated into telemedicine systems to support clinicians in monitoring patients’ emotional states during remote consultations. Similarly, in elderly care facilities, this technology may assist in detecting emotional changes and tailoring care strategies accordingly. Consequently, it is expected to contribute to extending the healthy life expectancy of Japanese older adults.

## Figures and Tables

**Table 1:** Examination of related works (emotion-recognition approaches).

Author	Techniques	Dataset	Targets	Features
Almeida et al. [19]	Xception	FER2013	Natural emotions	Facial expression
		SFEW		
Manalu et al. [22]	Custom CNN-RNN	Emognition Wearable Dataset 2020	Natural emotions	Facial expression
	InceptionV3-RNN			
	MobileNetV2-RNN			
Pradhan et al. [36]	HEPLM	WESAD	Natural emotions	ECG, EDA, EMG, RSP, skin temperature
Jaswal et al. [38]	GWO + CNN	Personal	Natural emotions	EEG, MFCC
Zhang et al. [40]	Mask R-CNN	Personal	Natural emotions	HR (noncontact)
				Eye state change
				HR (noncontact), Eye state change, Facial expression
Chatterjee et al. [41]	MobileNet	Thermal Face Database	Intentional emotions	Thermal images (skin temperature)
	MobileNet +GL-MFO			

**Table 2:** Types of emotions observed in this study.

Types of Emotions
Sympathy
Encouragement
Gratitude
Surprise
Impression
Admiration
High Praise
Amusement
Interest
Concern
Concentration
Disappointment
Sadness
Boredom

**Table 3:** Hyperparameters used in this study.

Hyperparameter	Value
Batch size	1024
Lookback	30 (one second)
Intermediate layer	100
Epoch	100
Loss function	Binary cross-entropy
Optimizer	Adam [56]
Number of features	11

**Table 4:** Macroaverage F1 score for each participant when assigned as test data.

Participants	F1 Score (%)
A	82.28
B	86.49
C	98.76
D	90.24
E	97.32
F	97.89
G	96.76
H	94.95
I	85.24
Average	92.21

**Table 5:** Comparative results of each method.

Author	Techniques	Dataset	Features	F1 Score (%)
The authors of the present study	BLSTM	Personal	Skin temperature and eye-opening degree	92.21
Almeida et al. [19]	Xception	FER2013	Facial expression	84.74
		SFEW		70.02
Manalu et al. [22]	Custom CNN–RNN	Emognition Wearable Dataset 2020	Facial expression	72.12
	Inception V3–RNN			71.34
	MobileNet V2–RNN			61.07
Pradhan et al. [33]	HEPLM	WESAD	ECG, EDA, EMG, RSP, and skin temperature	98.21
Jaswal et al. [35]	GWO + CNN	Personal	EEG and MFCC	98.08
Zhang et al. [37]	Mask R-CNN	Personal	HR (noncontact)	58.28
			Eye state change	83.10
			HR (noncontact), eye state change, and facial expression	84.99

## Data Availability

The data presented in this study are available upon request from the corresponding author due to privacy concerns regarding the study participants.

## Abbreviations

The following abbreviations are used in this manuscript:

ECG	Electrocardiogram
EMG	Electromyogram
EEG	Electroencephalography
HR	Heart Rate
GSR	Galvanic Skin Response
EDA	Electrodermal Activity
RSP	Respiration
EEG	Electroencephalogram
MFCC	Mel Frequency Cepstral Coefficient
GWO	Gray Wolf Optimization
GL-MFO	Grunwald–Letnikov Moth Flame Optimization
IRT	Infrared Thermography
ROIs	Regions of Interest
LT	Luminance Temperature
ATC	Amount of Temperature Change
EOD	Eye-opening Degree
BLSTM	Bidirectional Long Short-term Memory
